# Uncharged isocoumarin-based inhibitors of urokinase-type plasminogen activator

**DOI:** 10.1186/1472-6769-6-1

**Published:** 2006-02-08

**Authors:** Justin J Heynekamp, Lucy A Hunsaker, Thomas A Vander Jagt, Lorraine M Deck, David L Vander Jagt

**Affiliations:** 1 Department of Chemistry, University of New Mexico, Albuquerque, NM, USA; 2 Department of Biochemistry and Molecular Biology, University of New Mexico School of Medicine, Albuquerque, NM, USA

## Abstract

**Background:**

Urokinase-type plasminogen activator (uPA) plays a major role in extracellular proteolytic events associated with tumor cell growth, migration and angiogenesis. Consequently, uPA is an attractive target for the development of small molecule active site inhibitors. Most of the recent drug development programs aimed at nonpeptidic inhibitors targeted at uPA have focused on arginino mimetics containing amidine or guanidine functional groups attached to aromatic or heterocyclic scaffolds. There is a general problem of limited bioavailability of these charged inhibitors. In the present study, uPA inhibitors were designed on an isocoumarin scaffold containing uncharged substituents.

**Results:**

4-Chloro-3-alkoxyisocoumarins were synthesized in which the 3-alkoxy group contained a terminal bromine; these were compared with similar inhibitors that contained a charged terminal functional group. Additional variations included functional groups attached to the seven position of the isocoumarin scaffold. N- [3-(3-Bromopropoxy)-4-chloro-1-oxo-1*H*-isochromen-7-yl]benzamide was identified as an uncharged lead inhibitor of uPA, K_i _= 0.034 μM. Molecular modeling of human uPA with these uncharged inhibitors suggests that the bromine occupies the same position as positively charged arginino mimetic groups.

**Conclusion:**

This study demonstrates that potent uncharged inhibitors of uPA can be developed based upon the isocoumarin scaffold. A tethered bromine in the three position and an aromatic group in the seven position are important contributors to binding. Although the aim was to develop compounds that act as mechanism-based inactivators, these inhibitors are competitive reversible inhibitors.

## Background

Multiple proteases, including matrix metalloproteases (MMP-2, MMP-9 and MMP-14), cysteine proteases (cathepsin B and cathepsin L), aspartyl protease (cathepsin D) and serine proteases (plasmin, matriptase and urokinase) participate in cancer cell growth, metastasis and angiogenesis [1-4]. High expression of proteases often correlates with a poor prognosis [5,6]. Urokinase (uPA) plays an especially important role in extracellular proteolysis that contributes to cancer cell metastasis. Many cancer cells secrete pro-uPA and its receptor uPAR; binding of pro-uPA to uPAR leads to its activation, with subsequent generation of plasmin by the uPA-catalyzed hydrolysis of extracellular plasminogen [7,8]. The increased production of plasmin leads to degradation of extracellular matrix both by plasmin itself and by other proteases that are activated by plasmin. The surface location of bound uPA provides directionality to the degradation of matrix, thereby assisting the directional migration of cancer cells. uPA in complex with uPAR also affects other biological processes including signaling pathways that influence cell proliferation [9]. uPA has become a major target for development of non-peptidic small molecule inhibitors as potential anti-cancer drugs [10,11].

Most of the efforts to develop potent and selective inhibitors of uPA have focused on arginino mimetics based upon the trypsin-like specificity of uPA. The development of selective inhibitors of uPA is a challenge due to the large number of serine proteases with trypsin-like specificity, including factor VII, factor X and tissue-type plasminogen activator. Extensive structure-based drug development has provided potent and selective inhibitors of uPA; these generally are arginino mimetics with amidine or guanidine functional groups built onto aromatic or heterocyclic scaffolds (Figure 4) [12-16]. A major limitation to the use of these inhibitors is their poor bioavailability, which is at least partly owing to the presence of the positively charged amidine or guanidine group. This has limited clinical studies of these uPA inhibitors.

**Figure 1:**
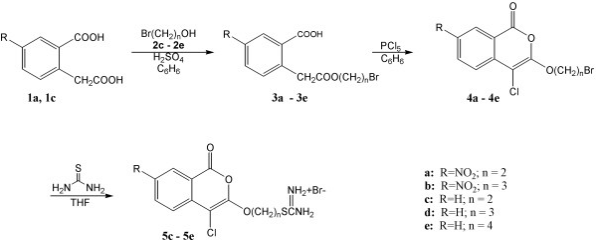
Scheme 1

**Figure 2:**
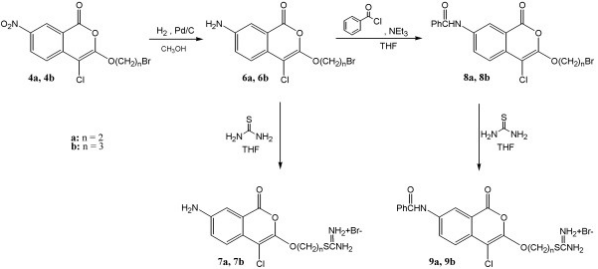
Scheme 2

**Figure 3:**
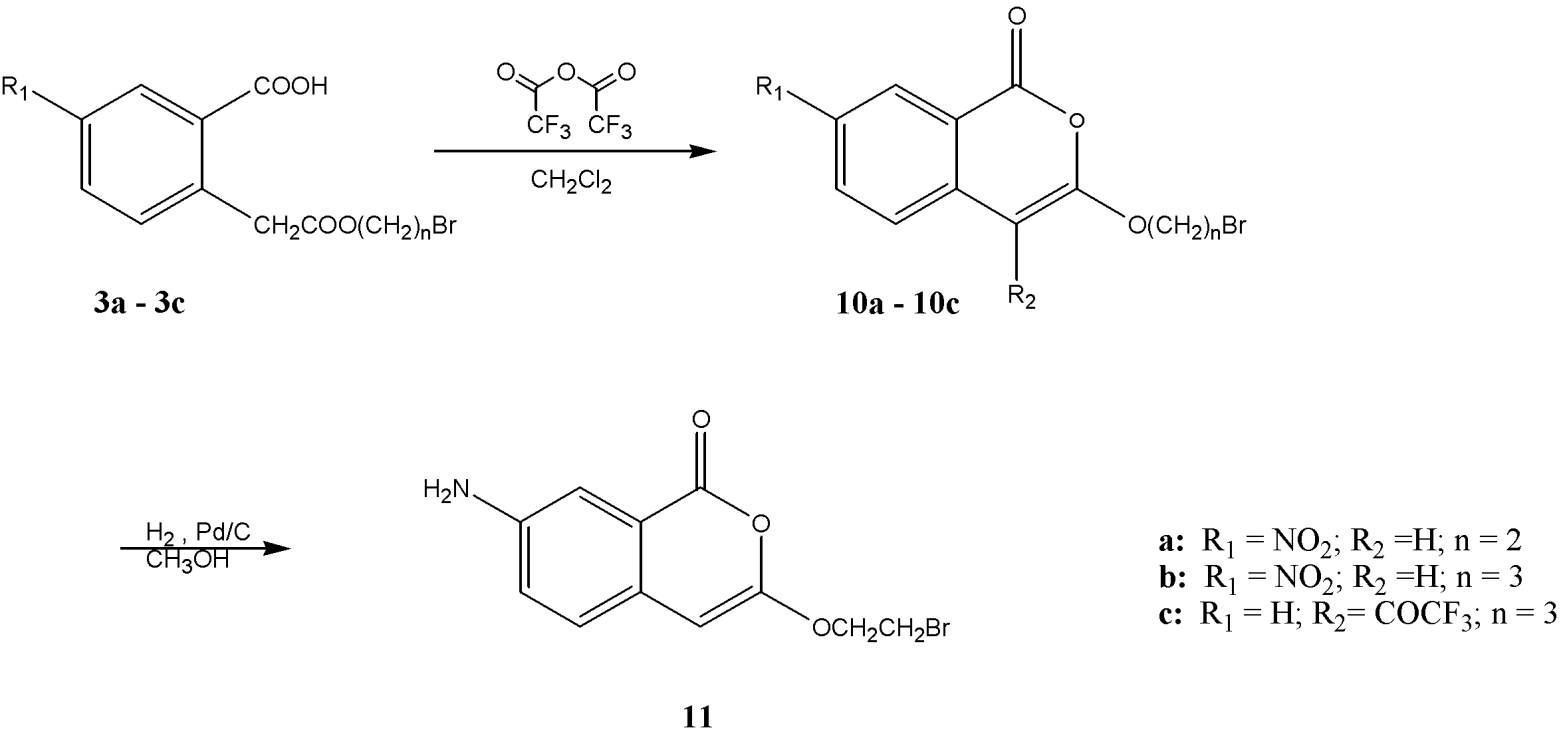
Scheme 3

**Figure 4:**
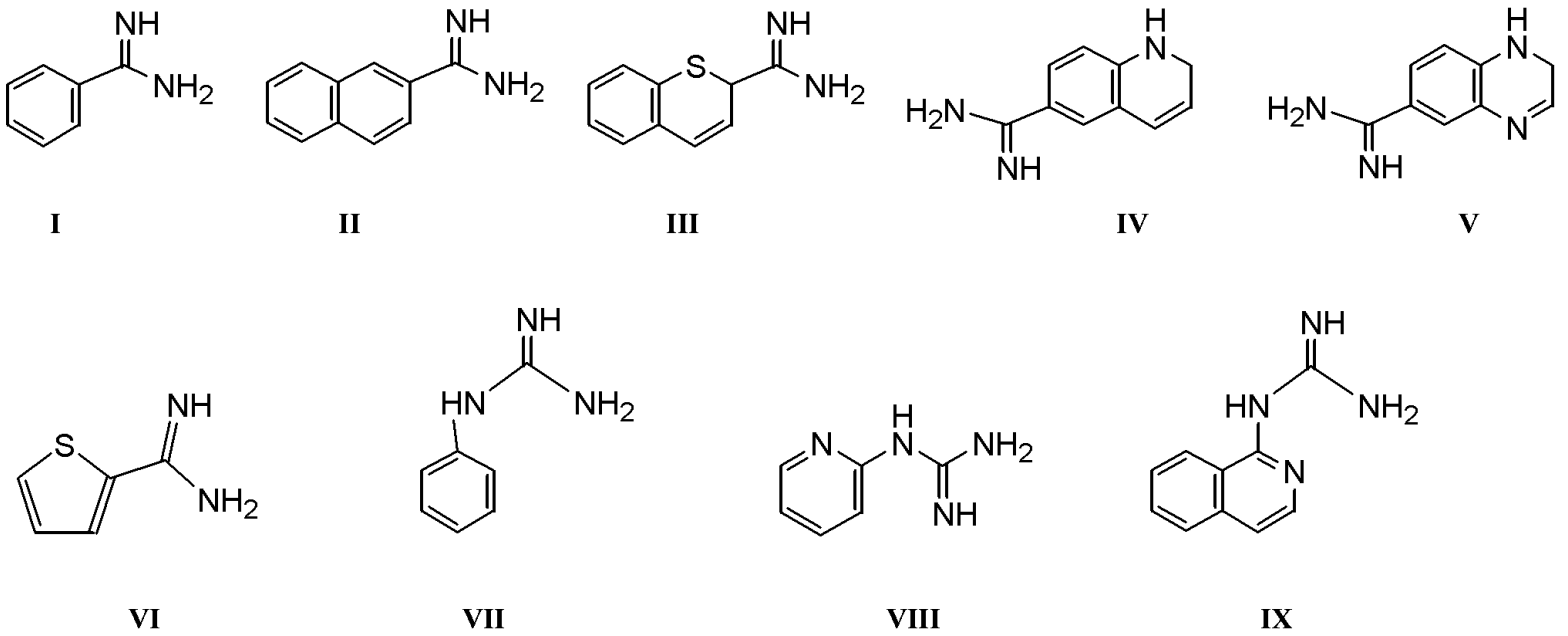
Scaffolds that have been utilized to develop arginino mimetic uPA inhibitors in the amidine (I-VI) and guanidine (VII-IX) series [12–16].

In the present study, we have focused on the synthesis and testing of uncharged compounds as leads for the development of uPA inhibitors with improved bioavailability. 4-Chloroisocoumarin was selected as the scaffold, in which substituted 3-alkoxy groups were introduced that contained neutral terminal functional groups or charged terminal functional groups [17]. Additional substituents were introduced into the seven position. 4-Chloroisocoumarin scaffolds have been used in studies of serine protease inhibitors, [17] but with limited application to uPA [18]. The choice of the 4-chloroisocoumarin scaffold was based upon the potential of these compounds to function as mechanism-based inactivators [17]. In this study we demonstrate that introduction of bromine in place of a terminal charged functional group in the 3-alkoxy substituent provides uncharged uPA inhibitors with low micromolar dissociation constants. Further introduction of substituents at the seven position of these uncharged uPA inhibitors provides compounds with low nanomolar dissociation constants. These inhibitors may serve as lead compounds for the development of new uPA inhibitors. Molecular modeling with human uPA suggests that the bromine occupies the same site as the arginino mimetic functional groups.

## Results and discussion

### Chemistry

Compounds **4a-4e**, which are 3-bromoalkoxy-4-chloroisocoumarins, were synthesized as shown in Figure 1. Two of the compounds, **4a **and **4b**, have a nitro group in position seven. These compounds have varying lengths of bromoalkoxy groups tethered in position three of the isocoumarin scaffold. 5-Nitrohomophthalic acid (**1a**) was prepared by regioselective nitration of homophthalic acid (**1c**) using fuming nitric acid [19]. 5-Nitrohomophthalic acid (**1a**) and homophthalic acid (**1c**) were monoesterified using bromoalcohols, compounds **2c-2e**, in the presence of sulfuric acid to give moderate yields of bromoesters, **3a-3e**. Monoesterification at the saturated acid has been attributed to the mesomeric effect of the carboxyl with the double bond in the aryl ring [20]. Cyclization of the esters, compounds **3a-3e**, with phosphorus pentachloride in toluene gave 3-bromoalkoxy-4-chloroisocoumarins, **4a-4e**, in moderate yields using a variation of a published method [17]. Compounds **4a-4e **were synthesized to test the importance of an uncharged group in the three position of 4-chloroisocoumarins and the length of the tether of the alkoxy group.

Compounds **5c-5e **are 3-isothioureidoalkoxy-4-chloroisocoumarin salts and were synthesized as shown in Figure 1. Nucleophilic substitution of the bromine in compounds **4c-4e **was achieved by refluxing these compounds with thiourea in tetrahydrofuran to give the hydrobromide salts, compounds **5c-5e**, in moderate yields. The 7-nitrosubstituted isocoumarins, compounds **4a **and **4b**, did not give any identifiable products when reacted with thiourea in tetrahydrofuran. Compounds **5c-5e **were synthesized to test the importance of a charged alkoxy group in the three position of 4-chloroisocoumarins.

The synthesis of 3-bromo-4-chloro-7-aminoisocoumarins, compounds **6a **and **6b**, is shown in Figure 2. These 7-aminoisocoumarins were prepared by reduction of 3-bromoalkoxy-4-chloro-7-nitroisocoumarins, compounds **4a **and **4b**, using hydrogen in the presence of a catalytic amount of 10% palladium on charcoal under pressure in methanol. Compounds **6a **and **6b **were synthesized to test the importance of an amino group in the seven position and an uncharged alkoxy group in the three position of 4-chloroisocoumarins. Nucleophilic substitution of the bromine in compounds **6a **and **6b **by reaction with thiourea afforded hydrobromide salts, **7a **and **7b**. Compounds **7a **and **7b **were synthesized to test the importance of a charged alkoxy group in the three position of 4-chloro-7-aminoisocoumarins.

3-Bromoalkoxy-4-chloro-7-benzamidoisocoumarins, compounds **8a **and **8b**, were synthesized by reaction of **6a **and **6b **with benzoyl chloride in the presence of triethylamine (Figure 2). Compounds **8a **and **8b **were synthesized to test the importance of an uncharged alkoxy group in the three position and a hydrophobic benzamide group in the seven position of 4-chloroisocoumarins. The two amides, **8a **and **8b **were reacted with thiourea to give the hydrobromide salts, **9a **and **9b**, in moderate yields. Compounds **9a **and **9b **were synthesized to test the importance of a charged alkoxy group in the three position and a hydrophobic benzamide group in the seven position of 4-chloroisocoumarins.

Figure 3 describes the synthesis of two 7-nitro-3-alkoxyisocoumarins, compounds **10a **and **10b**, which do not have a chlorine atom at position four. The synthesis of compound **10c**, which is a 3-alkoxy-4-trifluoroacetylisocoumarin, is also described in Figure 3. Cyclization of compounds **3a **and **3b, **which have a nitro group in position seven, using trifluoroacetic anhydride gave compounds **10a **and **10b**, which contain a hydrogen at position four. On the other hand, cyclization of **3c **which does not contain a nitro group in position seven gave compound **10c**, which contains a trifluoroacetyl group in position four. This may be attributed to the fact that the intramolecular cyclization of the initially formed enol is faster when there is resonance stabilization by the electron withdrawing nitro group. When the nitro group is absent the initially formed enol reacts with trifluoroacetic anhydride giving compound **10c**. Compounds **10a **and **10b **were synthesized to test the importance of a chlorine atom in position four and the presence of an electron withdrawing group in position seven of the isocoumarins. Compound **10c **gives information on the importance of a trifluoroacetyl group in position four of the isocoumarins.

Compound **11, **a 7-amino-3-alkoxyisocoumarin, was prepared by reduction of compound **10a **using hydrogen in the presence of a catalytic amount of 10% palladium on charcoal under pressure in methanol (Figure 3). Compound **11 **was synthesized to test the importance of a chlorine atom in position four and the presence of an electron donating group in position seven of the isocoumarins.

### Structure activity relationships

On the basis of docking studies using the crystal structure of human uPA [15] we examined whether the 4-chloroisocoumarin scaffold containing 3-alkoxy substituents is predicted to be a good template for the design of uPA inhibitors. Our interest focused on compounds having an uncharged bromine group in place of the charged arginino mimetic group at the terminal position of the 3-alkoxy group. Specifically, we compared the experimentally determined dissociation constants with the docking orientations predicted for compounds with the isocoumarin scaffold containing a charged isothiourea group or an uncharged bromine atom in the terminal 3-alkoxy position.

The first series of compounds compared the 4-chloroisocoumarin scaffold with 3-alkoxy substituents where the terminal functional group was an isothiourea group or bromine atom. The distance between the terminal group and the alkoxy oxygen was varied by the insertion of methylene units (Table 1). The designed inhibitors were shown to dock in the area of the active site containing the catalytic triad (serine 195, histidine 57, aspartic acid 102) and aspartic acid 189, which is the residue in trypsin-like enzymes that forms a salt bridge with the arginino mimetic groups. All of the compounds were predicted to reside in the active site with the thiourea group and the bromine atom in the S1-subsite which contains aspartic acid 189. Compounds **4c-4e**, which are 3-bromoalkoxy-4-chloroisocoumarins, are oriented in the active site with the uncharged bromine atom within 2.81–3.12 Å of aspartic acid 189. The best inhibitor in this series, compound **4d**, exhibited K_i _= 9 μM. Modeling of inhibitor **4d **with uPA also showed that three methylene units between the bromine and oxygen of the 3-alkoxy substituent provided the closest interaction between the isocoumarin carbonyl and the active site residue serine 195. Nevertheless, compound **4d **provided simple competitive inhibition with no evidence of rapid inactivation of uPA by **4d**. For comparison, compounds **5c-5e **were included in this series. These compounds contain the charged isothiourea group in place of the bromine atom and two, three or four methylene units between the isothiourea group and the oxygen atom. Modeling suggests that compounds **5c-5e **are all oriented in the active site with the charged isothiourea group forming a salt bridge with aspartic acid 189. The predicted distance between the isothiourea group and aspartic acid 189 for compounds **5c-5e **ranges from 2.49 – 2.58 Å. The best inhibitor, compound **5c**, exhibited a K_i _= 0.033 μM. Modeling suggested that the 3-alkoxy substituent with two methylenes between the isothiourea group and oxygen atom provided the closest interactions between the isocoumarin carbonyl and the active site serine. Clearly, the charged isothiourea group provides 3.5–4 kcal/mol more binding energy than the bromine atom in its interactions with aspartic 189 and surrounding residues. Thus, there is a large penalty in moving to the uncharged bromoalkoxy inhibitors. This may be the penalty that must be accepted if uncharged uPA inhibitors with improved bioavailability are to be developed in place of the charged uPA inhibitors that have been described [12-16].

**Table 1:** Dissociation constants for inhibition of human uPA

**Structure**	**Number**	**K_i _(μM)**	**Structure**	**Number**	**K_i _(μM)**
	4a	1.8 ± 0.2		7a	0.020 ± 0.003
	4b	2.4 ± 0.2		7b	0.038 ± 0.005
	4c	14 ± 1		8a	1.4 ± 0.2
	4d	9 ± 1		8b	0.034 ± 0.002
	4e	18 ± 2		9a	0.084 ± 0.007
	5c	0.033 ± 0.004		9b	0.010 ± 0.008
	5d	0.055 ± 0.003		10a	4.2 ± 0.4
	5e	0.14 ± 0.02		10b	4.3 ± 0.3
	6a	12 ± 1		10c	14 ± 2
	6b	9.5 ± 1		11	65 ± 9

**Figure 5:**
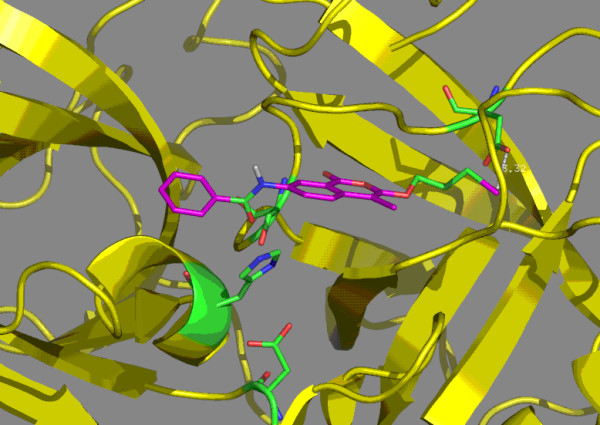
Molecular model of human uPA complexed with inhibitor **8b**. The bromine is within 3.32 Å of aspartic acid 189.

**Figure 6:**
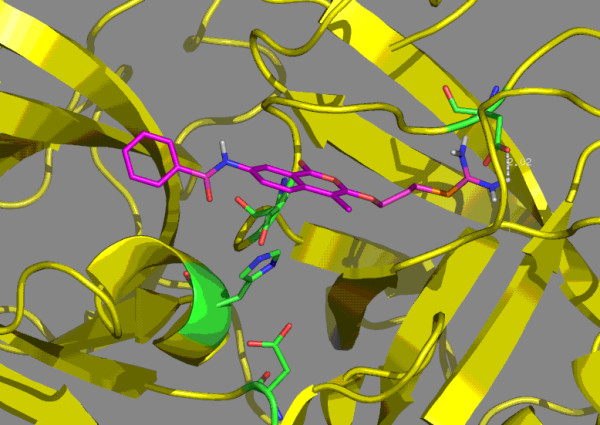
Molecular model of human uPA complexed with inhibitor **9b**. The isothiourea is within 2.02 Å of aspartic acid 189.

The second series of uPA inhibitors examined the effects of substituents in the seven position. Compounds **6a**, **6b**, **7a **and **7b**, which are 7-amino-4-chloroisocoumarins are attractive candidates because of the increased hydrogen bonding possibilities. Based upon modeling, compounds **6a **and **6b**, which have an uncharged bromine atom on the 3-alkoxy group, are within 2.80-3.15 Å of aspartic acid 189. Compounds **7a **and **7b**, which have a charged isothiourea group within 2.75–2.85 Å from aspartic acid 189. Compounds **8a**, **8b**, **9a **and **9b **are 7-substituted benzamides that were synthesized to investigate potential hydrophobic interactions from groups at the seven position. The uncharged bromine atoms of compounds **8a **and **8b **are within 2.93–3.32 Å of aspartic acid 189. The charged isothiourea group in compounds **9a **and **9b **are within 2.75 Å of aspartic acid 189. In the isothiourea series (**7a**, **7b**, **9a **and **9b**), the 7-substituted compounds did not exhibit any significant improvement in binding to uPA (Table 1). By comparison, the compounds in the bromo series with hydrophobic groups at the seven position (**8a **and **8b**) exhibited markedly improved binding; for **8b**, Ki = 0.034 μM, which represents a 300-fold improvement over **4d**. Inhibitor **8b **was the best inhibitor in the bromo series and can be viewed as a promising lead compound for development of uncharged inhibitors of uPA. The improvement that the benzamide group in **8a **and **8b **of the bromo series provided compared to the lack of improvement that the benzamide group provided in the isothiourea series may relate to increased flexibility. The bromo group has weak interactions with aspartic acid 189; this may allow the inhibitors in the bromo series to slide within the active site and S1 sub-site to orientations that allow the 7-benzamide group to find additional energetically favorable interactions. By comparison, the strong salt bridge in the uPA complexes with inhibitors in the isothiourea series may lock the inhibitors into the S1 sub-site. The modeled uPA complexes with inhibitors **8b **and **9b **are shown in Figures 5 and 6 respectively. The additional binding energy for **8b **appears to be the result of additional interactions of the aromatic ring of the benzamide group, especially with disulfide cysteine 58-cysteine 42.

The presence of a 7-nitro group also was beneficial. Compounds **4a **and **4b, **which are 3-bromoalkoxy-7-nitroisocoumarins, showed improved binding to uPA compared to the unsubstituted compounds. Compound **4b **which has three methylene units between the bromine atom and the oxygen of the 3-alkoxy group has a Ki = 2.4 μM, which is an 4-fold improvement over the unsubstituted compound **4d**.

Compounds **10a**, **10b **and **11 **were synthesized to determine whether the chlorine atom in position four contributed to binding. 3-Bromoalkoxy-7-substituted isocoumarins without a chlorine atom in the four position show dissociation constants that are about 2 fold higher compared to their counterparts that have a chlorine atom in the four position suggesting a modest role for the chlorine atom (Table 1). Compound **10c **which has trifluoroacetyl group in the four position did not show improved binding.

The isocoumarin-based inhibitors (Table 1) have the potential to function as suicide inhibitors or as substrates. However, simple reversible competitive inhibition was observed. The inhibitors were stable for several hours at neutral pH, as evidenced by no change in the spectral properties of the inhibitors. Addition of uPA for several hours at concentrations ten-fold higher than used in the kinetic studies did not produce any detectable changes in the inhibitors, suggesting that the inhibitors are not weak substrates of uPA. In addition, there was no loss of enzyme activity under these conditions, suggesting that the inhibitors are not functioning as suicide inhibitors. The observation of simple competitive inhibition is consistent with the modeling results in which the predicted orientations of the isocoumarin scaffold bound in the active site of uPA (figures 5 and 6) are not favorable for attack by serine 195.

## Conclusion

Inhibition of uPA by uncharged inhibitor **8b **represents a proof of concept that uPA inhibitors without a charged arginino mimetic group can be developed. Inhibitor **8b**, which exhibits a dissociation constant in the low nanomolar range comparable to those of known arginino mimetic inhibitors, represents a lead compound for future development of uncharged inhibitors of uPA. The present study did not address the issue of specificity. Many previous studies of uPA inhibitors with arginino mimetic groups attached to various scaffold have resulted in the development of selective inhibitors of uPA. This information should be useful for developing selective uncharged inhibitors of uPA.

## Methods

### Modeling

The x-ray crystal structure of human urokinase (pdb code 1EJN) was obtained from the protein data bank. All compounds shown in Table 1 were docked to the enzyme using Autodock 3.0 [21,22] on a cluster of Silicon Graphics workstations consisting of Octanes and O2s. The compounds were prepared using Sybyl 7.0 (Tripos Inc., St. Louis, MO). The molecules were drawn in, assigned partial charges using the included Gasteiger-Hückel method and energy minimized using the BFGS method. Minimizations were run for 10,000 iterations and the rotatable bonds defined before docking. The protein was prepared before docking in Sybyl by removing non-native substrates and water molecules. Polar hydrogens and Kollman Uni charges were added to the protein as well. The molecules were docked in an area defined around the active site serine 195 by a cube of 60 × 60 × 60 Å.

### Chemical synthesis

Reagent quality solvents were used without purification. Benzoyl chloride was distilled before use. Melting points were determined on a Thomas Hoover capillary melting point apparatus and are uncorrected. NMR spectra were recorded on a Bruker AC250 NMR spectrometer in CDCl_3 _unless noted. Chemical shifts are in ppm (δ) relative to TMS. High resolution mass spectra were recorded on a Waters/Micromass LCT- premier. Analytical data was obtained from Galbraith laboratories, Knoxville TN. 5-Nitrohomophthalic acid was prepared as reported [19]. Compounds **3a-3e**, **4a-4e**, **5c-5e**, **6a**, **6b**, **8a**, **8b 10a**, **10b **and **11 **were prepared according to published procedures [23]. Compounds **7a**, **7b**, **9a**, **9b **were prepared as reported [24].

### Kinetics

Human urokinase (Sigma/Aldrich, St. Louis, MO) and Spectrozyme UK (American Diagnostica, Stamford, CT) were used for the kinetic studies. Enzyme activity was routinely measured in 1 ml volumes of 0.1 M Tris, pH 8.8, Spectrozyme UK (10 μM to 150 μM) and 0.64 μg (3,770 units/mg protein) human urokinase. Reactions were monitored at 405 nm, 25°C, with a Perkin/Elmer Lambda S2 UV/vis spectrophotometer. Michaelis constants and K_i _values were determined from initial rate data, measured at 8 to 10 substrate concentrations, by non-linear regression analysis with SigmaPlot's Enzyme Kinetics Module™ (Chicago, IL, USA).

### Experimental

**2- [2-(2-Bromoethoxy)-2-oxoethyl]-5-nitrobenzoic acid (3a) **(66% yield) Tan crystals: mp 113–115°C (lit. [25] 90°C);^1^H NMR: δ 3.50 (t, 2H, *J *= 5.96 Hz) 4.21 (s, 2H) 4.43 (t, 2H, *J *= 6.06 Hz) 7.51 (d, 1H, *J *= 8.34 Hz) 8.39 (dd, 1H, *J *= 2.59 Hz, 8.39 Hz) 8.98 (d, *J *= 2.39 Hz);^13^C NMR: δ 28.40, 40.13, 64.17, 126.24, 126.45, 130.97, 133.32, 142.59, 146.84, 167.46, 169.63.

**2- [2-(3-Bromopropoxy)-2-oxoethyl]-5-nitrobenzoic acid (3b) **(51% yield) White crystals: mp 122–123°C; ^1^H NMR: δ 2.19 (m, 2H) 3.44 (t, 2H, *J *= 6.56 Hz) 4.18 (s, 2H) 4.28 (t, 2H, *J *= 5.96 Hz) 7.51 (d, 1H, *J *= 8.34 Hz) 8.39 (dd, 1H, *J *= 2.39 Hz, 8.35 Hz) 8.98 (d, 1H, *J *= 2.18 Hz) 9.78 (br s, 1H); ^13^C NMR: δ 29.18, 31.58, 40.51, 63.12, 126.84, 127.52, 129.77, 143.37, 147.47, 169.92, 170.05.

**2- [2-(2-Bromoethoxy)-2-oxoethyl]benzoic acid (3c) **(50% yield) Tan crystals: mp 82–83°C; ^1^H NMR: δ 3.50 (t, 2H, *J *= 6.25 Hz) 4.07 (s, 2H) 4.40 (t, 2H, *J *= 6.25 Hz) 7.27 (d, 1H, *J *= 7.75 Hz) 7.45 (t, 1H, *J *= 7.55 Hz) 7.54 (td, 1H, *J *= 1.39 Hz, 7.55 Hz) 8.14 (d, 1H, *J *= 7.74 Hz); ^13^C NMR: δ 28.50, 40.68, 64.08, 127.65, 128.32, 131.95, 132.43, 133.35, 136.40, 170.86, 172.43.

**2- [2-(3-Bromopropoxy)-2-oxoethyl]benzoic acid (3d) **(70% yield) White crystals: mp 79–80°C; ^1^H NMR: δ 2.16 (m, 2H, *J *= 6.31 Hz) 3.42 (t, 2H, *J *= 6.55 Hz) 4.05 (s, 2H) 4.24 (t, 2H, *J *= 5.96 Hz) 7.27 (d, 1H, *J *= 7.55 Hz) 7.40 (t, 1H, *J *= 7.65 Hz) 7.54 (td, 1H, *J *= 1.2, 7.35 Hz) 8.14 (dd, 1H, *J *= 0.99, 7.74 Hz); ^13^C NMR: δ 29.50, 31.80, 40.80, 62.52, 127.60, 128.35, 131.91, 132.41, 133.35, 136.86, 171.17, 172.47.

**2- [2-(4-Bromobutoxy)-2-oxoethyl]benzoic acid (3e) **A solution of 4-bromo-1-butanol (**2e**, 6.0 mL, 41.6 mmol), homophthalic acid (**1c**, 2.5 g, 13.8 mmol), and five drops of concentrated sulfuric acid was refluxed in benzene (50 mL) for four hours. The solution was cooled and washed with water (2 × 25 mL), brine (1 × 25 mL), and dried over magnesium sulfate. Filtration and evaporation of the solvent gave a dark oil that was triturated with hexane to afford a crude solid. Recrystallization from hexane/ethyl acetate gave 0.91 g (40%) of compound **3e **as white crystals: mp 84–86°C; ^1^H NMR: δ 1.79 (m, 2H) 1.87 (m, 2H) 3.38 (t, 2H, *J *= 6.45 Hz) 4.04 (s, 2H) 4.13 (t, 2H, *J *= 6.15 Hz) 7.27 (d, 1H, *J *= 7.55 Hz) 7.39 (t, 2H, *J *= 7.65 Hz) 7.54 (td, 1H, *J *= 1.4, 7.55 Hz) 8.13 (dd, 1H, *J *= 1.19, 7.74 Hz); ^13^C NMR: δ 27.33, 29.32, 33.19, 63.85, 127.56, 128.41, 131.88, 132.42, 133.33, 136.77, 171.32, 172.58.

**3-(2-Bromoethoxy)-4-chloro-7-nitro-1*H*-isochromen-1-one (4a) **(43% yield) Yellow crystals: mp 126–128°C (lit. [25] 120°C); ^1^H NMR: δ 3.67 (t, 2H, *J *= 6.16 Hz) 4.74 (t, 2H, *J *= 6.06 Hz) 7.86 (d, 1H, *J *= 8.94 Hz) 8.53 (dd, 1H, *J *= 2.39 Hz, 8.94 Hz) 9.03 (d, 1H, *J *= 2.38 Hz); ^13^C NMR: δ 27.72, 69.63, 90.86, 117.17, 123.81, 126.32, 129.82, 142.71, 145.47, 154.77, 157.06.

**3-(3-Bromopropoxy)-4-chloro-7-nitro-1*H*-isochromen-1-one (4b) **(76% yield) Pale yellow crystals: mp 131–134°C; ^1^H NMR: δ 2.37 (m, 2H) 3.59 (t, 2H, *J *= 6.26 Hz) 4.61 (t, 2H, *J *= 5.86 Hz) 7.81 (d, 1H, *J *= 8.94 Hz) 8.50 (dd, 1H, *J *= 2.09 Hz, 8.84 Hz) 8.99 (d, 1H, *J *= 1.79 Hz); ^13^C NMR: δ 28.49, 31.92, 68.56, 90.46, 116.93, 123.52, 126.22, 129.68, 142.77, 145.77, 155.34, 157.18.

**3-(2-Bromoethoxy)-4-chloro-1*H*-isochromen-1-one (4c) **(30% yield) Yellow solid: mp 81–82°C; ^1^H NMR: δ 3.65 (t, 2H, *J *= 6.35 Hz) 4.64 (t, 2H, *J *= 6.35 Hz) 7.41 (t, 1H, *J *= 7.15 Hz) 7.74 (m, 2H) 8.20 (d, 1H, *J *= 7.75 Hz); ^13^C NMR: δ 28.07, 69.37, 92.09, 117.53, 122.47, 126.55, 130.06, 135.62, 137.35, 152.08, 159.01.

**3-(3-Bromopropoxy)-4-chloro-1*H*-isochromen-1-one (4d) **(53% yield) Yellow crystals: mp 95–97°C; ^1^H NMR: δ 2.33 (m, 2H) 3.60 (t, 2H, *J *= 6.35 Hz) 4.51 (t, 2H, *J *= 5.76 Hz) 7.40 (t, 1H, *J *= 6.75 Hz) 7.72 (m, 2H) 8.19 (d, 1H, *J *= 7.94 Hz); ^13^C NMR: δ 28.88, 32.26, 68.28, 91.91, 117.58, 122.39, 126.43, 130.12, 135.62, 137.57, 152.74, 159.33.

**3-(4-Bromobutoxy)-4-chloro-1*H*-isochromen-1-one (4e) **A solution of **3e **(0.75 g, 2.3 mmol) and phosphorus pentachloride (1.23 g, 5.9 mmol) was refluxed in benzene (50 mL) for fourteen hours. The orange solution was cooled, washed with water (2 × 25 mL), saturated sodium bicarbonate (2 × 15 mL), brine (1 × 25 mL), and dried over magnesium sulfate. Filtration and evaporation of the solvent gave a yellow oil. Trituration with hexane gave 0.55 g (70%) of compound **4e **as white crystals: mp 75–77°C; ^1^H NMR: δ 1.98 (m, 2H) 2.06 (m, 2H) 3.48 (t, 2H, *J *= 6.25 Hz) 4.40 (t, 2H, *J *= 5.96 Hz) 7.38 (td, 1H, *J *= 1.59 Hz, 7.50 Hz) 7.70 (m, 2H) 8.17 (d, 1H, *J *= 7.55 Hz); ^13^C NMR: δ 27.33, 29.32, 33.12, 40.80, 63.81, 127.55, 128.41. 131.86, 132.38, 133.30, 136.76, 171.26, 172.29. Exact mass calcd for C_13_H_12_BrClO_3_: 329.9658, observed (M+H) 330.9734.

**2- [2-(4-Chloro-1-oxo-1*H*-isochromen-3-yloxy)ethyl]isothiourea hydrobromide (5c) **(64% yield) Yellow solid: mp 168–170°C (lit. [24] 167–169°C); ^1^H NMR: (DMSO-d_6_) δ 3.65 (t, 2H, *J *= 5.66 Hz) 4.58 (t, 2H, *J *= 5.67 Hz) 7.53 (t, 1H, *J *= 7.65 Hz) 7.69 (d, 1H, *J *= 8.14 Hz) 7.92 (t, 1H, *J *= 7.05 Hz) 8.13 (d, 1H, *J *= 7.75 Hz) 9.15 (br s, 4H); ^13^C NMR: δ 29.73, 68.11, 90.48, 117.18, 121.72, 126.70, 129.48, 135.99, 136.56, 152.18, 158.35, 169.11.

**2- [3-(4-Chloro-1-oxo-1*H*-isochromen-3-yloxy)propyl]isothiourea hydrobromide (5d) **(40% yield) Yellow solid: mp 159–163°C (lit. [24] 165–167°C);^1^H NMR: (DMSO-d_6_) 2.21 (m, 2H) 3.40 (t, 2H, *J *= 7.18 Hz) 4.55 (t, 2H, *J *= 6.11 Hz) 7.62 (td, 1H, *J *= 0.95, 7.60 Hz) 7.79 (d, 1H, *J *= 7.70 Hz) 8.02 (td, 1H, *J *= 1.23, 7.70 Hz) 8.23 (dd, 1H, *J *= 1.25, 7.50 Hz) 10.09 (br s, 4H); ^13^C NMR: δ 26.66, 28.53, 68.68, 90.43, 117.13, 121.66, 126.59, 129.47, 135.96, 136.66, 152.63, 158.53, 169.36.

**2- [4-(4-Chloro-1-oxo-1*H*-isochromen-3-yloxy)butyl]isothiourea hydrobromide (5e) **A solution of **4e **(0.25 g, 0.75 mmol) and thiourea (0.075 g, 0.98 mmol) in dry tetrahydrofuran (25 mL) was refluxed for forty-eight hours. The resulting pale yellow solid was filtered and washed with hot tetrahydrofuran (3 × 10 mL) to give 0.2 g (65%) of compound **5h **as a pale yellow solid: mp 160–162°C; ^1^H NMR: (DMSO-d_6_) δ 1.82 (br s, 4H) 3.24 (t, 2H, *J *= 6.45 Hz) 4.39 (t, 2H, *J *= 5.75 Hz) 7.50 (t, 1H, *J *= 7.45 Hz) 7.65 (d, 1H, 7.45 Hz) 7.89 (t, 1H, *J *= 7.25 Hz) 8.09 (d, 1H, *J *= 7.75 Hz) 9.07 (br s, 4H); ^13^C NMR: δ 24.92, 27.35, 29.59, 69.96, 90.20, 116.95, 121.56, 126.48, 129.45, 135.94, 136.73, 152.80, 158.59, 169.56.

**7-Amino-3-(2-bromoethoxy)-4-chloro-1*H*-isochromen-1-one (6a) **Compound **4a **(2.2 g, 6.3 mmol) was reduced on a Parr apparatus with hydrogen over 10% palladium on charcoal (50 mg) in ethanol (25 mL) until the reaction stopped absorbing hydrogen. The solution was filtered through celite and the filtrate was evaporated. The resulting crude solid was chromatographed (dichloromethane) to give 1.55 g (78%) of compound **6a **as yellow crystals: mp 134–136°C, (lit. [26] 134–137°C); ^1^H NMR: δ 3.63 (t, 2H, *J *= 6.46 Hz) 3.95 (br s, 2H) 4.56 (t, 2H, *J *= 6.36 Hz) 7.10 (dd, 1H, *J *= 2.58 Hz, 8.54 Hz) 7.43 (d, 1H, *J *= 2.58 Hz) 7.54 (d, 1H, *J *= 8.74 Hz); ^13^C NMR: δ 28.18, 69.87, 93.59, 113.09, 119.21, 123.54, 124.04, 128.24, 145.63, 149.90, 159.47.

**7-Amino-3-(3-bromopropoxy)-4-chloro-1*H*-isochromen-1-one (6b) **(75% yield) Yellow crystals: mp 106–107°C (lit. [24,26] 98–100°C); ^1^H NMR (DMSO-d_6_) δ 2.29 (m, 2H) 3.60 (t, 2H, *J *= 6.36 Hz) 4.42 (t, 2H, *J *= 5.76 Hz) 7.09 (dd, 1H, *J *= 2.09 Hz, 8.44 Hz) 7.42 (d, 1H, *J *= 1.99 Hz) 7.51 (d, 1H, *J *= 8.54 Hz); ^13^C NMR: δ 29.11, 32.36, 68.71, 93.35, 113.11, 119.17, 123.58, 123.91, 128.42, 145.56, 150.49, 159.74.

**2- [2-(7-amino-4-chloro-1-oxo-1*H*-isochromen-3-yloxy)ethyl]isothiourea hydrobromide (7a) **(40% yield) Pale yellow solid: mp d 150°C; ^1^H NMR: (DMSO-d_6_) δ 3.59 (br s, 2H) 4.47 (br s, 2H) 5.81 (br s, 2H) 7.21 (d, 1H, *J *= 8.94 Hz) 7.26 (s, 1H) 7.44 (br d, 1H) 9.11 (br s, 4H);^13^C NMR: δ 29.73, 68.11, 90.48, 117.18, 121.72, 126.70, 129.48, 135.99, 136.56, 152.18, 158.35, 169.11.

**2- [3-(7-Amino-4-chloro-1-oxo-1*H*-isochromen-3-yloxy)propyl]isothiourea hydrobromide (7b) **A solution of **6b **(0.25 g, 0.75 mmol), thiourea (0.071 g, 0.94 mmol) and tetrahydrofuran (25 mL) was refluxed for forty-eight hours to give a yellow precipitate. The precipitate was filtered and washed with hot tetrahydrofuran (3 × 25 mL), and recrystallized from methanol/ether to give 0.06 g (20%) of compound **7b **as a pale yellow solid: mp 173°C; (lit. [24] 160–162°C); ^1^H NMR: (DMSO-d_6_) δ 2.07 (br s, 2H) 3.30 (br s, 2H) 4.32 (br s, 2H) 7.16 (d, 1H, *J *= 7.94 Hz) 7.26 (s, 1H) 7.41 (d, 1H, *J *= 7.95 Hz) 9.05 (br s, 4H);^13^C NMR: δ 26.73, 28.35, 69.26, 92.87, 110.88, 118.81, 122.84, 123.11, 124.66, 148.23, 149.41, 159.06, 169.37.

**N- [3-(2-Bromoethoxy)-4-chloro-1-oxo-1*H*-isochromen-7-yl]benzamide (8a) **To a solution of **6a **(0.75 g, 2.4 mmol) in dry tetrahydrofuran (20 mL) was added benzoyl chloride (0.35 mL, 2.8 mmol) and triethylamine (0.33 mL, 2.3 mmol). The solution was stirred at room temperature for fourteen hours after which time the triethylamine hydrochloride was filtered off and washed with hot tetrahydrofuran (2 × 10 mL). The filtrate was evaporated to give a pale yellow solid that was recrystallized from tetrahydrofuran/hexane to afford 0.60 g (75%) of compound **8a **as a pale yellow solid: mp 214–216°C; ^1^H NMR: (DMSO-d_6_) δ 3.83 (t, 2H, *J *= 5.46 Hz) 4.65 (t, 2H, *J *= 5.46 Hz) 7.56 (m, 3H) 7.71 (d, 1H, *J *= 8.93 Hz) 7.99 (d, 2H, *J *= 8.15) 8.29 (dd, 1H, *J *= 2.39 Hz, 8.74 Hz) 8.68 (d, 1H, *J *= 2.18 Hz) 10.63 (s, 1H); ^13^C NMR: δ 30.49, 69.98, 91.08, 117.64, 119.23, 122.38, 127.52, 127.86, 128.20, 131.62, 131.90, 134.12, 137.90, 151.40, 158.35, 165.43. Exact mass calcd for C_18_H_13_BrClNO_4_: 420.9716, observed (M+H) 421.9788.

**N- [3-(3-Bromopropoxy)-4-chloro-1-oxo-1*H*-isochromen-7-yl]benzamide (8b) **(82% yield) Pale yellow solid: mp 193–194°C; ^1^H NMR: (DMSO-d_6_) δ 2.28 (m, 2H) 3.66 (t, 2H, *J *= 6.56 Hz) 4.44 (t, 2H, *J *= 5.96 Hz) 7.55 (m, 3H) 7.68 (d, 1H, *J *= 8.74 Hz) 7.98 (d, 6.56 Hz) 8.25 (dd, 1H, *J *= 1.99 Hz, 8.74 Hz) 8.66 (d, 1H, *J *= 1.98 Hz) 10.62 (s, 1H); ^13^C NMR: δ 30.74, 32.09, 69.13, 91.36, 118.01, 119.74, 122.80, 127.88, 128.43, 128.67, 133.10, 132.46, 134.47, 138.15, 152.20, 158.95, 165.96. Exact mass calcd for C_19_H_15_BrClNO_4_: 434.9873, observed (M+H) 435.9959.

**2- [2-(7-Benzamido-4-chloro-1-oxo-1*H*-isochromen-3-yloxy)ethyl]isothiourea hydrobromide (9a) **A solution of **8a **(0.3 g, 0.71 mmol) and thiourea (0.06 g, 0.78 mmol) in dry tetrahydrofuran (25 mL) was refluxed for twelve hours. The resulting pale yellow solids were filtered and washed with hot tetrahydrofuran (3 × 10 mL) to give 0.06 g (17%) of compound **9a **as a pale yellow solid: mp 173–175°C. Evaporation of the filtrate afforded **8a **(0.2 g). Yield based on recovered starting material is 51%; ^1^H NMR: (DMSO-d_6_) δ 3.68 (br s, 2H) 4.60 (br s, 2H) 7.59 (m, 3H) 7.74 (d, 1H, *J *= 8.54 Hz) 8.03 (d, 2H, *J *= 6.75 Hz) 8.32 (d, 1H, *J *= 8.14 Hz) 8.73 (s, 1H) 9.18 (br s, 4H) 10.69 (s, 1H); ^13^C NMR: δ 29.85, 68.32, 91.00, 117.72, 119.47, 122.53, 127.60, 128.22, 128.33, 131.79, 132.06, 134.12, 137.96, 151.44, 158.43, 165.63, 169.18.

**2- [3-(7-Benzamido-4-chloro-1-oxo-1*H*-isochromen-3-yloxy]propyl)isothiourea hydrobromide (9b) **(25% yield) Pale yellow solid: mp 203–204°C;^1^H NMR: (DMSO-d_6_) δ 2.12 (m, 2H) 3.31 (br s, 2H) 4.44 (t, 2H, *J *= 5.66 Hz) 7.56 (m, 3H) 7.71 (d, 1H, *J *= 8.74 Hz) 8.00 (d, 2H, *J *= 6.95 Hz) 8.28 (d, 1H, *J *= 8.54 Hz) 8.70 (s, 1H) 9.07 (br s, 4H) 10.65 (s, 1H); ^13^C NMR: δ 27.21, 28.79, 69.37, 91.38, 118.13, 119.85, 122.92, 128.04, 128.60, 128.77, 132.21, 132.57, 134.61, 138.33, 152.36, 159.05, 166.02, 169.83. Exact mass calcd for C_20_H_18_ClN_3_O_4_S: 431.0707, observed (M+H) 432.0780.

**3-(2-Bromoethoxy)-7-nitro-1*H*-isochromen-1-one (10a) **A solution of **3a **(1.5 g, 4.5 mmol) and trifluoroacetic anhydride (0.64 mL, 5.0 mmol) in dichloromethane (50 mL) was stirred at room temperature for sixteen hours. The solution was evaporated, washed with water (1 × 25 mL), saturated sodium bicarbonate solution (1 × 25 mL), dried over magnesium sulfate, and evaporated to afford 1.23 g (87%) of a crude yellow solid. Recrystallization from isopropanol gave 0.66 g (47%) of compound **10a **as yellow crystals: mp 95–97°C; ^1^H NMR: δ 3.65 (t, 2H, *J *= 5.96 Hz) 4.54 (t, 2H, *J *= 5.86 Hz) 5.77 (s, 1H) 7.42 (d, 1H, *J *= 8.74 Hz) 8.38 (d, 1H, *J *= 8.54 Hz) 8.96 (s, 1H); ^13^C NMR: δ 27.51, 68.87, 81.12, 117.12, 125.70, 126.17, 129.28, 144.97, 145.11, 158.81, 160.02.

**3-(3-Bromopropoxy)-7-nitro-1*H*-isochromen-1-one (10b) **(55% yield) Yellow crystals: mp 117–118°C; ^1^H NMR: δ 2.37 (m, 2H) 3.59 (t, 2H, *J *= 6.26 Hz) 4.38 (t, 2H, *J *= 5.86 Hz) 5.72 (s, 1H) 7.43 (d, 1H, *J *= 8.74 Hz) 8.41 (dd, 1H, *J *= 2.48 Hz, 8.84 Hz) 9.02 (d, 1H, *J *= 2.19 Hz);^13^C NMR: δ 28.80, 31.46, 67.29, 80.02, 117.02, 125.55, 126.07, 129.15, 144.79, 145.21, 158.94, 160.89.

**3-(3-Bromopropoxy)-4-trifluoroacetyl-1*H*-isochromen-1-one (10c) **A solution of **3d **(0.60 g, 2.0 mmol) and trifluoroacetic anhydride (0.38 mL, 2.7 mmol) in dichloromethane (25 mL) was stirred at room temperature for fourteen hours. The solution was evaporated and the oil was chromatographed (chloroform) to afford 0.45 g (59%) of compound **10c **as white crystals: mp 116–117°C; ^1^H NMR: δ 2.38 (m, 2H) 3.54 (t, 2H, *J *= 6.26 Hz) 4.70 (t, 2H, *J *= 5.96 Hz) 7.42 (m, 1H) 7.74 (m, 1H) 8.10 (d, 1H, *J *= 8.34 Hz) 8.22 (d, 1H, *J *= 7.95 Hz); ^13^C NMR: δ 28.24, 31.37, 68.94, 90.90, 115.81, 116.01, 123.40, 126.75, 130.26, 135.87, 136.27, 157.73, 162.08, 179.97. Anal. Calcd. for C_14_H_10_BrF_3_O_4_: C, 44.35; H, 2.66. Found: C, 44.25; H, 2.99.

**7-Amino-3-(2-bromoethoxy)-1*H*-isochromen-1-one (11) **A solution of **10a **(1.5 g, 4.7 mmol) in methanol/ethyl acetate (1:1, 25 mL) was reduced on a Parr apparatus with hydrogen and 10% palladium on charcoal. After the reaction stopped absorbing hydrogen it was filtered through celite and the celite was washed with methylene chloride (3 × 50 mL). The filtrate was evaporated to near dryness keeping the temperature below 40°C. The semisolid was recrystallized from methylene chloride/methanol to afford 1.10 g (81%) of compound **11 **as yellow crystals: mp > 280°C; ^1^H NMR: (DMSO-d_6_) 3.79 (t, 2H, *J *= 5.17 Hz) 4.36 (t, 2H, *J *= 4.97 Hz) 5.50 (s, 2H) 5.82 (s, 1H) 7.03 (d, 1H, *J *= 8.54 Hz) 7.19 (m, 2H); ^13^C NMR: δ 30.30, 68.71, 80.35, 110.20, 117.91, 123.17, 125.84, 128.03, 147.16, 154.71, 160.45.

## Authors' contributions

JJH conducted the synthetic chemistry with the assistance of LMD. LAH and TAV conducted the kinetic studies. DLVJ and LDM conceived the study and wrote the manuscript with the assistance of JJH.
